# Supplementary material for: Metabolic Remodeling with Hepatosteatosis Induced Vascular Oxidative Stress in Hepatic ERK2 Deficiency Mice with High Fat Diets
Source: Int J Mol Sci. 2022 Jul 31;23(15):8521. doi: 10.3390/ijms23158521 (PMC9369278; doi:10.3390/ijms23158521)
Supplement: Supplementary file 1 [file ijms-23-08521-s001.zip › ijms-1819576-Supplemental Table.pdf]

Supplemental Table S1: Comparison of Tissue Weights

	NC		HFHSD	
	**Control**	**LE2KO**	**Control**	**LE2KO**
Liver (mg)	1040.6±12.3	1047.2±12.5	1270.3±45.8	1622.2±90.2#
Kidney (mg)				
Right	182.9±5.9	188.0±4.6	204.6±7.3	197.3±4.9
Left	177.5±4.9	176.6±5.1	194.2±6.2	199.6±4.8
Heart (mg)	136.8±4.3	135.3±3.3	143.0±3.4	148.9±3.4
Epidydimal fat (mg)	389.3±101.1	415.8±47.7	1305.6±83.6	1257.7±31.6
Perirenal fat (mg)	222.7±20.8	239.3±15.8	660.5±22.4	645.7±3.7
Spleen (mg)	77.0±3.6	73.1±3.2	102.7±4.8	120.1±6.9

Data are mean ± SD. # $p < 0.05$ vs. HFHSD-Control. n = 8 in all groups.

Supplemental Table S2: Serum Amino Acid Profile

	NC		HFHSD		p Value
	Control	LE2KO	Control	LE2KO	
Taurine	662.7±211.2	552.4±144.7	492.5±107.8	590.4±108.9	0.17
Aspartic acid	4.1±1.2	3.2±0.4	4.4±0.9	7.6±3.4	0.004
Hydroxyproline	13.7±4.3	14.4±7.1	TR	TR	-
Threonine	118.0±18.8	110.4±17.6	89.1±12.2*	84.4±12.8	0.0003
Serine	79.1±18.3	76.3±23.5	64.4±8.4	84.7±24.7	0.24
Asparagine	33.6±4.4	32.2±4.6	27.9±4.9	29.1±5.2	0.09
**Glutamic acid**	**22.3±3.7**	**22.0±1.9**	**25.8±3.2**	**37.3±9.1#**	**<0.0001**
Glutamine	528.9±83.1	551.8±79.1	510.6±41.8	567.8±56.6	0.36
**α-Aminoadipic acid**	**7.0±3.5**	**5.3±2.6**	**3.8±0.5**	**4.4±0.3#**	**0.06**
Proline	64.7±10.6	62.6±15.6	52.3±8.1	51.9±8.8	0.05
Glycine	161.6±58.4	159.7±58.7	134.4±17.1	111.2±21.8	0.09
Alanine	223.5±47.7	217.7±91.7	176.9±23.7	181.8±55.2	0.30
Citruline	33.7±6.2	31.7±9.4	30.7±6.4	42.6±10.7#	0.03
α-Aminobutyric acid	20.1±6.6	20.0±7.3	13.8±4.8	6.0±1.1#	<0.0001
Valine	187.7±47.3	162.7±23.2	127.3±27.6*	140.4±43.8	0.02
Cystine	11.5±2.9	10.9±3.0	11.0±3.0	12.0±3.3	0.88
Methionine	36.7±8.1	35.5±6.1	31.7±4.9	31.4±5.6	0.25
Isoleucine	92.5±27.3	81.4±15.0	60.4±13.6*	66.2±19.2	0.01
Leucine	163.3±54.7	142.0±33.3	104.6±23.0*	109.4±29.1	0.01
Tyrosine	45.2±10.8	40.8±4.6	44.6±9.2	61.1±12.7#	0.002
Phenylalanine	68.0±10.8	60.9±8.5	49.5±4.2*	53.0±9.7	0.0009
**Monoethanolamine**	**13.5±2.7**	**12.7±2.1**	**12.2±1.2**	**15.8±2.1#**	**0.01**
Histidine	60.7±13.1	60.1±6.5	50.8±4.8	58.2±6.7	0.09
3-Methylhistidine	11.7±1.9	11.8±1.8	13.8±1.1*	13.9±1.0	0.004
**Tryptophan**	**52.6±5.8**	**50.2±8.1**	**53.6±12.7**	**73.7±16.0#**	**0.0009**
**Ornithine**	**41.0±13.9**	**35.5±9.1**	**32.0±15.5**	**76.2±26.7#**	**0.0003**

Lysine	199.9±39.3	185.2±50.9	136.5±11.9*	155.8±30.0	0.006
**Arginine**	**70.8±12.8**	**62.7±15.2**	**44.3±10.9***	**27.9±19.9**	**0.0002**
**Ornithine / Arginine ratio**	**0.56 (0.49-0.66)**	**0.57 (0.53-0.64)**	**0.59 (0.50-0.64)**	**2.74 (1.2-10.2)#**	**0.001**

Data are mean ± SD, median (interquartile range). (nmol/mL). Final column reflects overall group differences. * $p < 0.05$ vs. NC-Control; #p<0.05 vs. HFHSD-Control. ND n = 8 in all groups.
